# Mobile Robot-Based Gait Training after Total Hip Arthroplasty (THA) Improves Walking in Biomechanical Gait Analysis

**DOI:** 10.3390/jcm10112416

**Published:** 2021-05-29

**Authors:** Eric Röhner, Anke Mayfarth, Christian Sternitzke, Frank Layher, Andrea Scheidig, Horst-Michael Groß, Georg Matziolis, Sabrina Böhle, Klaus Sander

**Affiliations:** 1 Orthopaedic Professorship of the University Hospital Jena, Orthopedic Department of the Waldkliniken Eisenberg, 07607 Eisenberg, Germany; f.layher@waldkliniken-eisenberg.de (F.L.); g.matziolis@waldkliniken-eisenberg.de (G.M.); s.boehle@waldkliniken-eisenberg.de (S.B.); k.sander@waldkliniken-eisenberg.de (K.S.); 2 Tediro GmbH, Ehrenbergstr. 11, 98693 Ilmenau, Germany; anke.mayfart@tediro.com (A.M.); christian.sternitzke@tediro.com (C.S.); 3 TU Ilmenau, Neuroinformatics and Cognitive Robotics Lab, PF 100565, 98684 Ilmenau, Germany; andrea.scheidig@tu-ilmenau.de (A.S.); fg-nikr@tu-ilmenau.de (H.-M.G.)

**Keywords:** mobile robots, rehabilitation robots, physiotherapy, motion capture, digital therapies

## Abstract

There are multiple attempts to decrease costs in the healthcare system while maintaining a high treatment quality. Digital therapies receive increasing attention in clinical practice, mainly relating to home-based exercises supported by mobile devices, eventually in combination with wearable sensors. The aim of this study was to determine if patients following total hip arthroplasty (THA) could benefit from gait training on crutches conducted by a mobile robot in a clinical setting. Method: This clinical trial was conducted with 30 patients following total hip arthroplasty. Fifteen patients received the conventional physiotherapy program in the clinic (including 5 min of gait training supported by a physiotherapist). The intervention group of 15 patients passed the same standard physiotherapy program, but the 5-min gait training supported by a physiotherapist was replaced by 2 × 5 min of gait training conducted by the robot. Length of stay of the patients was set to five days. Biomechanical gait parameters of the patients were assessed pre-surgery and upon patient discharge. Results: While before surgery no significant difference in gait parameters was existent, patients from the intervention group showed a significant higher absolute walking speed (0.83 vs. 0.65 m/s, *p* = 0.029), higher relative walking speed (0.2 vs. 0.16 m/s, *p* = 0.043) or shorter relative cycle time (3.35 vs. 3.68 s, *p* = 0.041) than the patients from the control group. Conclusion: The significant higher walking speed of patients indicates that such robot-based gait training on crutches may shorten length of stay (LOS) in acute clinics. However, the number of patients involved was rather small, thus calling for further studies.

## 1. Introduction

Total hip arthroplasty (THA) is one of the most effective orthopedic surgeries to improve quality of life. Most candidate patients suffer from end-stage osteoarthritis that severely affects their walking abilities. Ageing populations and a growing number of overweight subjects contribute to an increase in THA demand in most countries worldwide. Regaining a mobility level post-surgery that comes close to the patients’ previous mobility as a baseline is the goal. It is commonly accepted that physiotherapy after THA has a positive impact on muscle strength, range of motion, pain, or gait which all impact patient mobility [1]. Physiotherapy starts shortly after operation, primarily as inpatient therapy in the hospital where surgery took place [2,3]. It may be further organized as inpatient therapy in rehabilitation clinics, as outpatient therapy, home therapy, or combinations thereof.

Multiple studies provide empirical insights into efforts in reducing costs in the healthcare system while maintaining treatment quality. This includes, among others, earlier starts of the therapy, such as on the day of operation vs. on the first post-operative day [2,3,4,5], (costly) inpatient vs. (cheaper) outpatient therapy after hospital discharge [6], or home-based (cheaper) vs. outpatient (more expensive) physiotherapy [7].

In this light, several digital therapies have been studied on patients following lower limb surgery. In general, digital therapies often allow enhancing existing therapy programs by providing support by substitutive or during additional training to enhance patient mobility. Many of these approaches also allow reducing costs in the healthcare system, so there is a dual function here. Exemplary studies in this light are Wijnen et al. who investigated the effect of a web-based mobile app that provided training instructions, measuring physical activity by a necklace motion sensor [8,9]. Dias Correia and Nogueira used a similar approach involving three motion sensors for a tablet-based home-based therapy after total knee arthroplasty [10]. Robot usage is particularly rare apart from applications in neurology where therapies address trunk instability, using devices like the Lokomat, a (stationary) treadmill allowing patient fixation and computer-controlled therapy programs, or the Andago, a mobile, self-propelled frame with physical patient support in which the mobile function substitutes the treadmill (both made by Hocoma, Volketswil, Switzerland). An exception is the work of Vox et al. who devised a therapy program with the NAO robot (Softbank Robotics, Tokyo, Japan) in front of a stationary Kinect2 3D camera (Microsoft, Redmond, WA, Unit States) connected to a computer system that processes images of patients [11] like the motion sensor data processing as mentioned above for mobile apps [8,9,10]. While the robot solely animated the patients, the actual analyses were done with the computer system, capturing, among others, speed or symmetry of movements, extension/flexion of the angles (as e.g., defined by thresholds), the number of repetitions of the exercises, while the robot then provided feedback, e.g., about continuing or stopping the training [11]. Overall, digital therapies not only allow standardization of treatment in an environment in which very diverse views on patient treatment exist, it also allows shifting therapist interventions from e.g., monotonous tasks towards more complex ones.

In this study, a mobile robot for gait training on crutches was used that operated in an acute clinic, offering walking exercises in three-point gait starting on the second post-operative day until patient discharge on the, on average, sixth post-operative day. Given that the anterolateral approach is the predominant form of THA surgery in Germany, and Diagnosis-related Group (DRG) statistics, discharge occurs 8.3 days post-surgery on average (with I47C as dominant DRG). This robot made use of its on-board sensors and algorithms to capture the patient, identified eventual gait errors, and gave instructions to correct these errors.

The focus on gait training stems from various observations in the literature that walking during physiotherapy has a positive impact on walking speed (e.g., Hesse et al. with their additional treadmill training for overweight THA patients in an acute clinic or Heiberg et al. with a special walking exercise program for outpatient physiotherapy) [12,13]. As walking speed may contribute to earlier discharge, it may represent a way to further reduce length of stay and save healthcare system costs. The present study, however, held patient discharge constant and assessed patient mobility outcomes. In its discussion, the present work also points into directions in which robot-based gait training may make further contributions, such as addressing the treatment of gait imbalances and training motivation.

## 2. Methods

### 2.1. Technical System Specification

**Figure 1:**
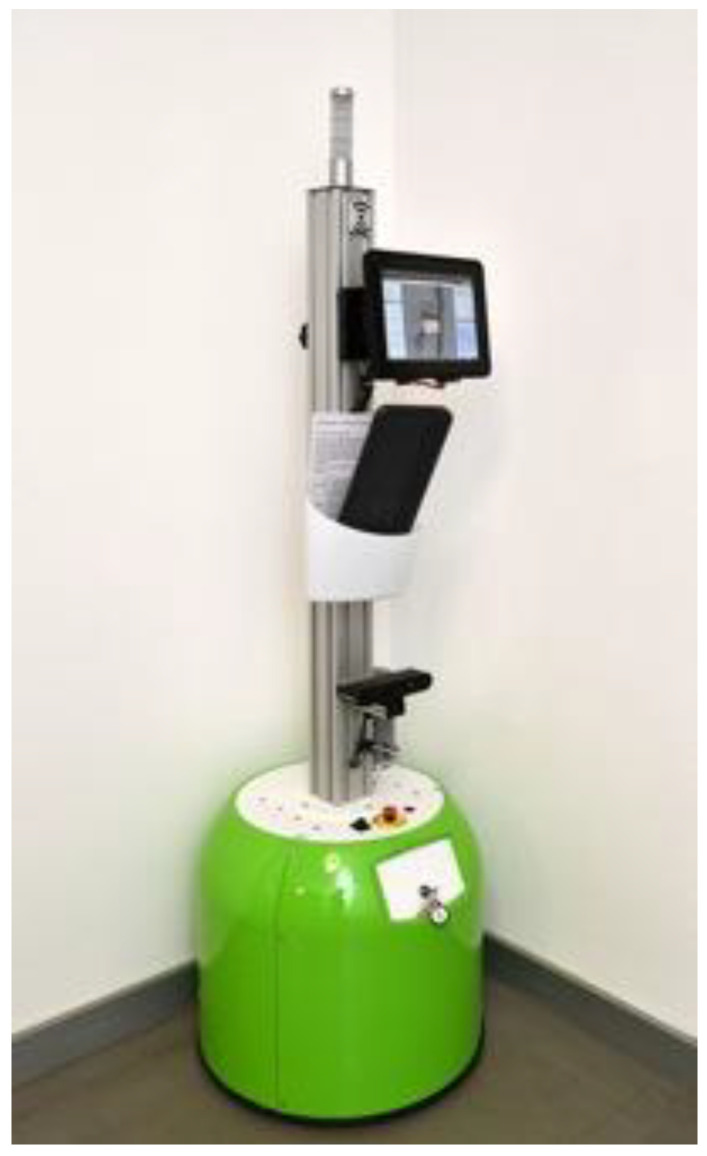
The mobile robotic system based on the MetraLabs Tory platform.

The mobile robotic system was based on the Tory platform (MetraLabs, Ilmenau, Germany) allowing a speed of up to 1 m/s (Figure 1). Movements of the patient were captured by an RGB-3D camera (Orbbec Astra Pro, Troy, MI, United States). This camera is integrated in the robot, with the help of which space-time parameters of walking (step length, stance duration), joint angles of the lower extremities and the upper body inclination can be extracted. The positions of the forearm crutches are also recorded in the 3D image.

**Figure 2:**
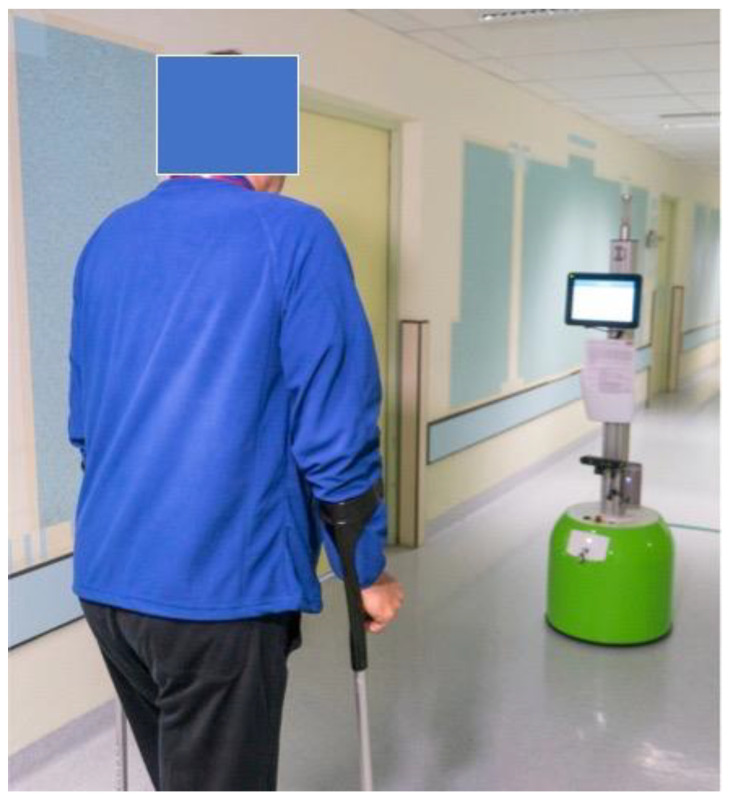
A patient after hip arthroplasty using crutches during the gait training with the robot.

The patients with robot-assisted gait training had to register with an RFID chip on the robot and completed a training course of initially 5 min from the 2nd to the 6th postoperative day, which could be increased up to 10 min. At the beginning, the physiotherapist gave a short introduction in the handling/use of the robot. In the further course, the patients trained independently with the robot that analyzed the gait based on previously classified parameters like stance duration, step-length, the position of the crutches relative to the feet. Using the knowledge of several physiotherapists, rules and threshold values for typical errors when walking on crutches had been determined and implemented beforehand. Based on these rules and thresholds, errors made by the patients during gait training could be identified by the robot and corrected by using a speech output module) (Figure 2).

### 2.2. Instrumental 3-D Gait Analysis

The gait analysis was carried out using the Vicon 3D movement analysis system (Vicon, Oxford, UK) with 10 infrared cameras (Bonita 10, frame rate 200 Hz, company, Vicon, Oxford, UK) and three force plates (one Kistler—Kistler Instrumente AG, Winterthur, Switzerland and two AM¬TI—AMTI, Watertown, MA, USA; 1000 Hz each). The program “Nexus V.2.8.2” was available for data acquisition and processing and the program “Polygon V.4.1” (Vicon, Oxford, UK) was available for evaluation. According to a selected model, the patients are provided with markers at distinctive body points. These are recorded by the infrared cameras while walking and the 3D location coordinates of the markers are saved.

**Figure 3:**
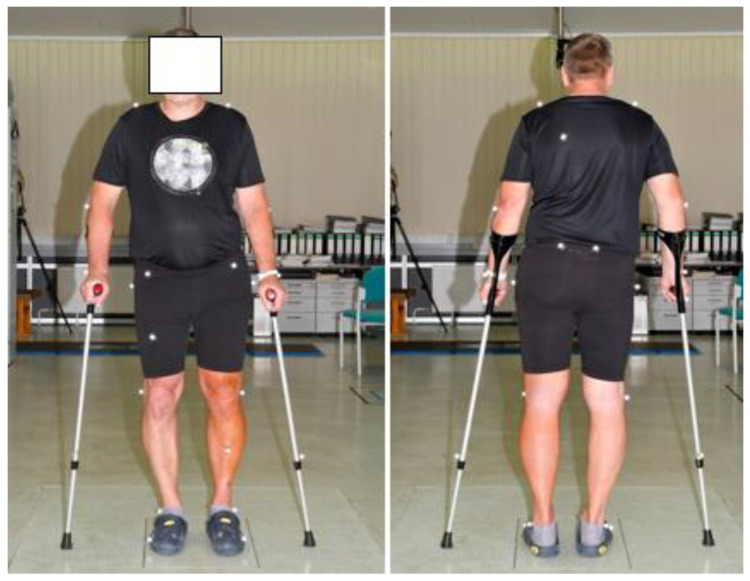
A patient with markers. A whole-body model was used to record the upper body movement in addition to the lower extremities.

A whole-body model (“Eisenberger model”) was used to record the upper body movement in addition to the lower extremities [14]. For this purpose, the patients were provided with 21 markers, e.g., at the joint pivot points and the pelvis. Since the use of the crutches was necessary for the postoperative measurement, the forearm crutches were each affixed with two additional markers. The arrangement of the markers is shown in Figure 3.

A double step is analyzed using a gait analysis (phase between two heel strikes on the same leg). This step cycle is divided into the stance phase (heel strike of one leg until toe detachment) and the swing phase (toe detachment until heel strike). The stance phase comprises around 60% of the gait cycle. In order to be able to directly compare the right and left leg, the values are normalized, i.e., the values for the stance phase of both legs are superimposed [15].

For the metrological recording of the walking, the patients paced an approx. 12 m long, level walking distance at a self-selected speed preoperatively without and postoperatively with crutches. There were 10 repetitions of measurements.

The time-distance parameters and also the angles from the temporal curve progressions (maximum and minimum values, ranges), which were used for the evaluation, resulted from the averaging of the 10 measurement runs. Due to the relieving effect of the forearm crutches, a load analysis (floor reaction forces; joint moments) was dispensed with. Thus, only time-distance and kinematic parameters were analyzed:

Time-distance parameters (also relative values, based on body size) [16]:

- Cadence
- Double support
- Cycle time
- Stance phase
- Stride length
- Walking speed.

Kinematic (force-independent) parameters:

- Joint angle (knee and hip joint) absolute + range (sagittal, frontal, transversal)
- Absolute torso and pelvic angles + range (sagittal, frontal, transversal)

#### Clinical Scores (PROMSs)

The following clinical questionnaires were determined preoperatively and postoperatively for both groups:

- Harris hip score (hip score)
- Q-5D-5L (quality of life)
- WOMAC (Western Ontario—McMaster Universities)
- VAS (visual analog scale)

### 2.3. Study Design

Following total hip arthroplasty thirty patients were included in this prospective study. Fifteen patients received the conventional physiotherapy program (control group) in the clinic (including 5 min of gait training supported by a physiotherapist). The robot group of 15 patients passed the same standard physiotherapy program, but the 5-min gait training supported by a physiotherapist was replaced by 2 × 5 up to 10 min of gait training conducted by the robot. Length of stay of the patients was set to five to six days (which is shorter than the mean length of stay of 8.3 days in Germany). Biomechanical gait parameters of the patients were assessed pre-surgery and upon patient discharge. All patients underwent the same surgical technique (anterolateral approach) for primary hip arthroplasty and, as is the standard program post-surgery in all clinics in Germany, had to walk on crutches.

### 2.4. Inclusion and Exclusion Criteria

Patents were included fulfilling the following criteria:

- Coxarthrosis as cause of THA
- BMI < 35
- Age 50–75 years
- Signed written consent form

Patients with the following criteria were excluded:

- Gait-affecting impairments (Morbus Parkinson, spinal diseases, hip dysplasia, etc.)
- Mentally impaired patients (e.g., dementia)
- Patients in malpractive litigation
- Patients in Malpractice Litigation

### 2.5. Statistical Analysis

The statistical analyses were conducted with IBM SPSS Version 19 (IBM, Armonk, NY, United States) and Stata 15 (Stata Corp., College Station, TX, United States). Tests on normal distributions were conducted with the Shapiro Wilk test. As not all parameters showed a normal distribution, the Mann-Whitney-*U*-Test for independent samples was employed. A * *p* value of <0.05 was defined as significant, *** *p* value of <0.001 was defined as highly significant.

## 3. Results

The aim was to achieve a comparable strength per study group. After consistent application of the inclusion and exclusion criteria such as diagnosis, primary total hip arthroplasty, age, secondary diseases, the robot group was reduced from originally 26 patients to 17 patients, and the control group from the original 21 to 20 patients. After two patients dropped, eight men and seven women were finally included in the robot group, whereas after a drop-out of five patients, five men and 10 women were finally included the control group. Dropout reasons were postponing surgery, withdrawal of consent, and transfer to another hospital post-surgery).

The average age of the robot group was 61.0 ± 6.4 years (max. 75.3 years and min. 50.6 years) and the average BMI was 29.5 ± 4.0 kg/m^2^ (max. 34.6 kg/m^2^ and min. 22.1 kg/m^2^). Nine patients were operated on the right and six on the left hip. All patients walked in the 3-point gait postoperatively.

In the control group, the average age was 63.9 ± 3.7 years (max. 69.9 years and min. 57.5 years). The average BMI was 30.3 ± 4.7 kg/m^2^ (max. 35.5 kg/m^2^ and min. 19.1 kg/m^2^). 4 patients were operated on the right and 11 on the left side. Ten patients walked in 3-point gait and 5 in 2-point gait postoperatively.

Both groups (robot and control) did not differ significantly in terms of age (*p* = 0.141) and BMI (*p* = 0.494), which allows a comparison. This was also reflected in the preoperative gait analysis parameters in which there were no differences between the robot and the control group. (Table 1, Table 2 and Table 3).

### 3.1. Time-Distance Parameters

Even giving the small sample size, patients from the robot group showed significantly better values post-operatively for the relative cadence (higher), the relative cycle time (shorter) and the walking speed (faster). This is an indication that gait training using robots had greater effects in terms of a faster gait than conventional gait training. * *p* < 0.05 (Table 2).

### 3.2. Range and Joint Angle Gradients

In the robot group there were highly significant better values of the relative range of sagittal knee flexion angles (higher) and range 2 of the sagittal knee flexion angle (swing phase) postoperatively, * *p* < 0.05. For both parameters, the range of motion improved significantly in the robot group compared to the control group (Table 3). There were no differences of trunk movement in the sagittal and frontal planes but a significant increase in the range of trunk rotation, *** *p* < 0.001.

### 3.3. Functional Scores

The evaluation of functional scores showed no significant differences for all scores between the robot group versus control group pre- and postoperatively (Table 4).

## 4. Discussion

While the benefits of physiotherapy after THA are generally acknowledged [1], and it frequently takes place in acute care clinics [17,18], there is a lack of consensus on the exact therapy program and only little evidence from controlled clinical trials, as a systematic review of the literature that appeared after 2008 [19] has shown. Other reviews and meta-analyses confirmed this view [19,20,21]. This variance in therapy program composition also leads to multiple divergent results in the literature, confirming or rejecting the usefulness of certain forms of therapy (e.g., home-based, outpatient or inpatient) in relation to any other [7,22]. In the light of highly divergent views on treatment methods, robot-based gait training, as other forms of digital therapies, may contribute to therapy standardization.

Walking as a component of the therapy program analyzed in this work was only examined in detail in one study mentioned in the reviews [19,20,21,22], namely in Heiberg et al.’s study [13]. In their controlled trial, after THA patients received a walking skill training program, including various exercises (for five minutes each) like sit to stand, single-leg stand, stairclimbing, walking, etc. This training program took place over six weeks with two sessions per week. The study found that patients who received that training program were 8% faster in the six-minute walk test (6 MWT) twelve months after surgery than the control group without any physiotherapy. The study by Heiberg et al. [13] differs from the present study in two important aspects: a) it was a therapy program following patient discharge from the acute clinic, and b) the control group did not receive physiotherapy at all. In the present study, the baseline physiotherapy program consisted of 20 min of daily physiotherapy involving hands on techniques like manual therapy, exercise training, lymphatic drainage including gait training on crutches for around five minutes and was conducted in an acute clinic starting on the first postoperative day.

The improvement in walking speed of more than 25% observed in the present study can be assessed in terms of its magnitude in view of Heiberg et al. [23]. It measured the mobility of patients after THA three and twelve months post-surgery based on the six minute walk test (6 MWT), thus indirectly measuring walking speed, finding that the score increased by about nine percent after three months and 28% after twelve months (in both cases pre-surgery as a baseline). This improvement in walking speed is in the same magnitude measured in the present study within four days of walking exercises, which is surprising given the few days of robot training.

There remain several implications from the findings of the present study in view of the literature: First, total hip arthroplasty means that gait mechanics may not necessarily return completely to normal, even almost one year after surgery [24]. The reason for this seems to be that pain-avoidance strategies may still be in place and these compensations have resulted in gait abnormalities that have not been fully resolved by physiotherapy [25]. These compensation strategies lead to an increased risk of falling, so Judd et al. argue that re-establishing normal gait patterns must be a core element of physiotherapy following THA [26,27]. Others also suggest that patients after THA have a higher incidence of falling, which decreases over time [28]. This indirectly indicates the necessity of improved training. With this in mind, the results from the present study imply that the robot-based gait training may help overcome impairments caused by musculoskeletal imbalances and reduce the risk of a later fall.

Second, Ewen et al. also found that patients after THA have lower hip ROM and walk more slowly than healthy subjects [29]. They attributed these effects to a lower level of patient motivation to regain full mobility. Interestingly, several studies of mobile assistance robots showed that these are perceived as companions vis-à-vis computer systems or smartphones, based on an effect known as personalization of embodiment. This effect leads to a higher training motivation [30,31,32,33]. In this light, the robotic system used in the present study may help improve training motivation and thus contribute to a higher level of mobility.

Third, improving patient mobility should contribute to earlier patient discharge. Other studies have investigated the effect of length of stay in clinics depending on the starting day of physiotherapy, comparing the day of operation versus the first post-operative day, finding that starting at the day of operation reduces length of stay [2,3,4,5]. Given the effect size of walking speed found in the present study, the robot-based gait training on crutches may contribute to shorter LOS. As length of stay has been decreasing during the past decade and continues to do so, given e.g., advances in surgical techniques and shifts from inpatient to outpatient physiotherapy, robot-based gait training as presented in this work may make a contribution particularly in those countries which still show longer hospitalization rates.

## 5. Limitations and Future Research

Apart from the small sample size, the biomedical engineers who conducted the assessment were not blinded and were also responsible for the statistical analysis. Blinding and separating assessments and analysis would have increased the validity of the results. Further, the 3D camera was not able to identify the crutches at all times, eventually leading to a bias in gait correction announcements provided by the robot. Future research should address these limitations. It would also be helpful to use multivariate analyses, including general physical activities as a control variable. The outcomes to be measured should not only include the WOMAC or Harris Hip Score but also frequently found measures of walking distance or speed, including the 6 MWT, the stairclimbing test, and eventually the TUG (timed-up-and-go-test). Further studies on robot intervention may also start on the day of operation. Future work could also test the effects of mobile robot-based gait training in patients on crutches after total knee arthroplasty (TKA) and other forms of lower limb surgery. In addition, follow-up assessments of the outcomes could be undertaken, e.g., 3 month and 12 months post-surgery.

Beaulieu et al. compared biomechanical gait patterns of THA patients one year post-surgery with those of healthy subjects, finding that walking speed is significantly lower due a shorter stride length, with smaller hip peak extension and flexion as well as hip extension/flexion ROM, and lower hip peak adduction [25]. These clinical findings were also broadly confirmed in a meta-study by Bahl et al. involving more than 2000 observations [24]. Interestingly, the increase in walking speed in the present study was based on a larger cadence, while the stride length of patients with and without robot treatment was the same. It would be interesting to investigate whether the advance of faster walking would diminish shortly after therapy or whether it would translate into long-term benefits that would eventually translate into changes in the stride length. If not, walking patterns of patients might change post-THA towards higher speed at shorter stride length (with the shorter stride lengths being obviously common for post-THA subjects). This would be an interesting consequence to investigate.

## 6. Conclusions

This work presented a novel way of gait training for patients walking on crutches following THA, involving robotic intervention. A significant higher walking speed in patients following robot-based gait-training was found. This may contribute to an increase in activities of daily living, increasing patient safety when crossing roads, etc. However, the number of patients involved was rather small, thus calling for further studies. 

Reflecting upon the literature, there remain a few areas where such robot-based gait-training may make contributions in the future: the reduction of gait imbalances, the increase in patient motivation during training (e.g., as a mediating effect), and eventual influences on the length of stay in clinics as a consequence of higher patient mobility for patients that underwent THA surgery based on the anterolateral approach. In addition, robot-assisted gait training, as other forms of digital therapies, may contribute to more standardization in treatment procedures which, according to the authors’ experience, vary widely among clinics.

## Figures and Tables

**Table 1:** Demonstrates basic patient parameters. *p*-values based on *U*-test.

	Control			Robot			Control vs. Robot *p*
	Mean	Min.	Max.	Mean	Min.	Max.	
Sex = female	10	-	-	7	-	-	0.277
Age	63.9	57.5	69.9	61.0	50.6	75.3	0.147
BMI	30.3	19.1	35.5	29.5	22.1	34.6	0.494
Operated side = left	10	-	-	7	-	-	0.277

**Table 2:** Shows the time and distance parameters of the gait analysis. A *p* value of <0.05 was defined as showing a significant difference.

	Control		Robot		Control vs. Robot	
	Pre	Post	Pre	Post	Pre	Post
	Median	Median	Median	Median	*p*	*p*
**Time and distance parameters**						
Cadence (steps per minute)	114.03	80.15	109.05	86.04	0.367	0.089
Cadence_relative (steps per minute)	0.78	00.54	0.76	00.59	0.567	0.041 *
Double support (s)	0.25	00.39	0.29	00.38	0.089	0.935
Double support_relative (s)	0.60	00.91	0.69	00.92	0.067	0.902
Cycle_time (s)	1.06	1.48	1.10	1.39	0.305	0.106
Cycle_time_relative (s)	2.61	3.68	2.67	3.35	0.567	0.041 *
Stance phase (%)	61.44	61.99	61.61	62.18	0.744	0.902
Stride length (m)	0.63	00.58	0.59	00.58	0.624	0.285
Stride length_relative (m)	0.37	00.33	0.35	00.33	0.233	0.775
Walking speed (m/s)	1.18	00.65	1.07	00.83	0.412	0.029 *
Walking speed_relative (m/s)	0.29	00.16	0.26	00.20	0.345	0.041 *

* *p* < 0.05.

**Table 3:** Shows ranges (in °) of the gait analysis. A *p* value of <0.05 was defined as showing a significant difference, and a difference with a *p* value of <0.001 was considered highly significant.

Ranges	Control		Robot		Control vs. Robot	
	Pre	Post	Pre	Post	Pre	Post
	Median	Median	Median	Median	*p*	*p*
Knee sagittal range	59.37	51.87	62.08	57.28	0.902	0.000 ***
Knee sagittal range1	5.31	4.25	6.46	6.78	0.285	0.367
Knee sagittal range2	47.07	41.96	47.14	45.48	0.653	0.026 *
Hip sagittal range	29.62	24.39	28.15	25.98	0.202	0.461
Trunk transversal range	17.65	12.14	20.36	15.58	0.367	0.006 *
Trunk frontal range	10.58	4.73	9.48	4.59	0.345	0.967
Trunk sagittal range	9.95	8.98	10.81	9.96	0.412	0.567
Pelvic tilt range	6.75	6.41	6.98	8.50	0.683	0.106
Pelvic oblique range	4.86	4.27	4.68	4.81	0.935	0.148
Pelvic rotational range	12.07	11.04	12.26	12.34	0.217	0.653
Hip frontal range	5.61	6.24	6.08	6.57	0.345	1.000

* *p* < 0.05, *** *p* < 0.001.

**Table 4:** Shows the functional scores. No significant differences were shown.

Functional Scores	Normal	Robot	*p*
	Median		
EQ-5D-5L-Index pre	0.72	0.76	0.775
EQ-5D-5L-Index post	0.75	0.79	0.081
VAS pre	7.00	5.25	0.325
VAS post	2.00	2.00	0.683
Pain pre (WOMAC)	5.60	4.40	0.152
Stiffness pre (WOMAC)	6.00	6.00	0.981
Function pre (WOMAC)	5.94	5.71	0.683
Global index pre (WOMAC)	5.48	5.48	0.614
Pain post (WOMAC)	1.80	1.60	0.744
Stiffness post (WOMAC)	2.50	3.00	0.902
Function post (WOMAC)	5.18	3.38	0.126
Global index post (WOMAC)	3.37	2.53	0.325
HHS pre	53.00	59.50	0.187
HHS post	58.00	60.50	0.389
